# Comparison of the Hemagglutination Inhibition Titers against Influenza Vaccine Strains in Japan from the 2017/2018 to 2021/2022 Seasons Using a Single Set of Serum Samples

**DOI:** 10.3390/v14071455

**Published:** 2022-06-30

**Authors:** Naruhito Otani, Kazuhiko Nakajima, Kaori Ishikawa, Kaoru Ichiki, Yoshiko Yoda, Takashi Ueda, Yoshio Takesue, Takuma Yamamoto, Susumu Tanimura, Masayuki Shima, Toshiomi Okuno

**Affiliations:** 1 Department of Public Health, Hyogo Medical University, Nishinomiya 663-8501, Japan; yoda-y@hyo-med.ac.jp (Y.Y.); shima-m@hyo-med.ac.jp (M.S.); 2 Department of Infection Control and Prevention, Hyogo Medical University, Nishinomiya 663-8501, Japan; nakajima@hyo-med.ac.jp (K.N.); i-kaori@hyo-med.ac.jp (K.I.); ichiki@hyo-med.ac.jp (K.I.); taka76@hyo-med.ac.jp (T.U.); takesuey@hyo-med.ac.jp (Y.T.); 3 Department of Legal Medicine, Hyogo Medical University, Nishinomiya 663-8501, Japan; tk-yamamoto@hyo-med.ac.jp; 4 Department of Public Health Nursing, Mie University Graduate School of Medicine, Tsu 514-0001, Japan; aruminat@gmail.com; 5 Department of Microbiology, Hyogo Medical University, Nishinomiya 663-8501, Japan; tmokuno@hyo-med.ac.jp

**Keywords:** influenza A (H1N1), influenza A (H3N2), influenza B/Victoria lineage, vaccine strain, cross-immunity, serology

## Abstract

In Japan, inactivated influenza vaccines are used. We measured titers of antibodies to vaccine strains of three influenza types—influenza A (H1N1), influenza A (H3N2), and influenza B/Victoria—from the 2017/2018 to 2021/2022 seasons, but not for influenza A (H3N2) from the 2018/2019 season, using a single set of serum samples from 34 healthy volunteers, and assessed the consistency in antibody positivity between seasons. The antibody titers in the 2017/2018 season were used as a reference. The influenza A (H1N1) antibody titer in 2019/2020 did not differ significantly from that in the 2017/2018 season, but the titers varied in the two subsequent seasons. The influenza A (H3N2) antibody titers toward the 2019/2020, 2020/2021, and 2021/2022 seasonal viruses differed significantly from that in the 2017/2018 season. The influenza B/Victoria antibody titer toward the 2019/2020 seasonal antigen differed from that in the 2017/2018 season, and the antibody positivity was inconsistent between seasons; however, the antibody titer in the 2020/2021 season did not differ significantly from those in the prior two seasons, and the antibody positivity was consistent between seasons. Antibody titers and their consistency can be used to evaluate cross-immunity of antibodies.

## 1. Introduction

In Japan, no seasonal influenza epidemic occurred in the 2020/2021 or 2021/2022 seasons [1,2]. This is considered to have been due to the effect of the COVID-19 pandemic and associated control measures.

Seasonal influenza is caused by the following four strains: influenza A (H1N1), influenza A (H3N2), influenza B/Yamagata, and influenza B/Victoria. In the three seasons before the COVID-19 pandemic, influenza B/Yamagata was the predominant influenza type in Japan, but epidemics of influenza A (H3N2) and influenza A (H1N1) were also observed in the 2017/2018 season [3]. In the 2018/2019 season, influenza A (H3N2) was the most prevalent, followed by influenza A (H1N1) and influenza B/Victoria [4]. In the 2019/2020 season, influenza A (H1N1) was the most prevalent, followed by influenza B/Victoria [5]. As the epidemic strains vary from season to season and are unpredictable, polyvalent vaccines are used [6].

Trivalent inactivated vaccines against two influenza A strains and an influenza B strain were used until the 2014/2015 season, but quadrivalent inactivated vaccines against the two influenza A subtypes and two influenza B lineages have been used in Japan since the 2015/2016 season. The vaccine strains used in each season since the 2017/2018 season are shown in Table 1. Of the four influenza vaccine strains, two to three have been changed each year since the 2017/2018 season [7].

The hemagglutination inhibition (HAI) titer is used as the gold standard for measuring seropositivity [8]. Immunological evaluation of vaccines is conducted using criteria based on HAI according to the European Medicines Agency (EMA) guidance [9]. Therefore, the antibody titers to vaccine strains in different age groups are evaluated each year using HAI [10,11,12,13]. However, as these results [10,11,12,13] are obtained using samples collected from different individuals each year, rather than from the same individuals, we considered that it is necessary to use the same serum for comparison of antibody titers against each vaccine antigen.

In this study, the antibody titers to vaccine antigens from the 2017/2018 to 2021/2022 seasons (excluding those to the 2018/2019 H3N2 antigen) were assessed and compared in a single set of serum samples, and the consistency of antibody positivity across seasons was assessed to investigate cross-reactivity.

## 2. Materials and Methods

### 2.1. Study Population and Vaccines

Thirty-four healthy adults (16 males and 18 females aged 29–63 years) were enrolled in this study, and their blood samples were collected between September 2017 and March 2018. The blood samples were obtained before vaccination in the 2017/2018 season. All the participants had been vaccinated in the 2016/2017 season. History of influenza infection was not known for any of the participants. The blood samples were centrifuged, and the serum samples were stored in two separate tubes at −80 °C until testing. One sample set was used to measure antibodies to influenza type A and the other was used to measure antibodies to influenza type B. Antibody titers to vaccine strains from the 2017/2018 to 2021/2022 seasons were measured using these serum samples. This study was approved by the Ethics Committee of Hyogo Medical University (protocol number: 1592).

### 2.2. Antibody Titration

The HAI antibody titers to the vaccine strains of each season were measured. After each sample was treated with receptor-destroying enzyme, it was diluted 1:10, and the amounts of HAI antibodies in the serum were measured using an influenza virus HAI test (Denka Seiken Co., Tokyo, Japan). The final dilution of the sample at which hemagglutination was completely inhibited was regarded as the HAI antibody titer. The HAI antibody titers were determined by a commercial laboratory (SRL, Inc., Tokyo, Japan). HAI antibody titers ≥1:40 were considered positive and those <1:40 were considered negative [8,9].

### 2.3. Evaluated Influenza Virus Types and Strains

The vaccine types and strains evaluated are shown in Table 1. In the 2018/2019 season, influenza A (H3N2) was not measured. The H1N1 antigen in 2018/2019 was the same as that in the 2017/2018 season, and the B/Victoria antigen in 2018/2019 was the same as that in the 2019/2020 season. As the vaccine strain of influenza B/Yamagata has not been changed since the 2017/2018 season, we only measured antibodies to the Phuket/3073/2013 strain.

### 2.4. Statistical Analyses

The statistical significance of changes in the HAI titer since the 2017/2018 season was assessed using the Wilcoxon signed-rank test, adjusting the *p*-value using the Holm method for paired titers, and Spearman’s rank correlation analysis was conducted for comparing the antibody titers between seasons. Next, the results were dichotomized into positive (≥1:40) or negative (<1:40), and the consistency of paired antibody titer results was assessed using the McNemar test, adjusting the *p*-value using the Holm method. All analyses were conducted using R version 4.1.3 (R Core Team, Vienna, Austria) [14].

## 3. Results

### 3.1. Changes in Vaccine Strains and Antibody Titers

The correlation coefficient of the antibody titers against influenza A (H1N1) compared with those in the 2017/2018 and 2019/2020 seasons was high (0.90), but the correlation coefficients for the comparison of titers in subsequent seasons with those in the 2017/2018 season decreased with each change in subtype (2020/2021 season vs. 2017/2018 season: 0.50; 2021/2022 season vs. 2017/2018 season: 0.38) (Table 2). No significant difference was observed in the antibody titers in the 2017/2018 and 2019/2020 seasons (*p* > 0.99), but significant differences were observed between the 2017/2018 and 2020/2021 seasons (*p* = 0.002) and between the 2017/2018 and 2021/2022 seasons (*p* = 0.014) (Table 3).

The correlation coefficients of the antibody titers against influenza A (H3N2) in the 2019/2020, 2020/2021, and 2021/2022 seasons compared with that in the 2017/2018 season were 0.59, 0.71, and 0.70, respectively (*p* < 0.01) (Table 2). Significant differences were also observed in the antibody titers between the 2019/2020 and 2020/2021 seasons, between the 2019/2020 and 2021/2022 seasons, and between the 2020/2021 and 2021/2022 seasons (*p* < 0.01).

The correlation coefficients of the antibody titers against influenza B/Victoria in the 2019/2020 and 2020/2021 seasons compared with that in the 2017/2018 season were 0.91 and 0.83, respectively (Table 2). A significant difference was observed between the 2017/2018 and 2019/2020 seasons (*p* < 0.001), but no significant difference was observed between the 2017/2018 and 2020/2021 seasons (*p* = 0.17) (Table 3).

### 3.2. Changes in Vaccine Strains and Positivity Rate of Hemagglutination Inhibition Titer

The positivity rate is shown in Table 4, the results of the McNemar test regarding the consistency are shown in Table 5, and the geometric mean titer (GMT) of each antibody is shown in Table 6.

The antibody titers against influenza A (H1N1) were positive in 5/34 (15%) samples in both the 2017/2018 and 2019/2020 seasons and in 1/34 (3%) samples in the 2020/2021 and 2021/2022 seasons (Table 4). The consistency of influenza A (H1N1) antibody positivity (≥1:40) did not differ significantly between the 2017/2018 and subsequent seasons (Table 5). The GMT decreased progressively (Table 6).

The proportion of samples that were antibody-positive against influenza A (H3N2) decreased from 20/34 (59%) in the 2017/2018 season to 3/34 (9%) in the 2019/2020 season, increased to 29/34 (85%) in the 2020/2021 season, but decreased again to 2/34 (6%) in the 2021/2022 season (Table 4). There was no significant difference in the consistency of antibody positivity between the 2019/2020 and 2021/2022 seasons (data not shown). Additionally, the positivity rate was high in the 2017/2018 and 2020/2021 seasons, but a significant difference was observed in the consistency (*p* < 0.01). The GMT was the highest (58.9) in the 2020/2021 season (Table 6).

The antibody-positivity rate against influenza B/Victoria increased from 12/34 (35%) in the 2017/2018 season to 22/34 (65%) in the 2019/2020 season, but decreased to 9/34 (26%) in the 2020/2021 season (Table 4). The antibody titers in the 2017/2018 and 2019/2020 seasons differed significantly (*p* < 0.001), but no significant difference was observed compared with that in the 2020/2021 season (*p* = 0.17) (Table 3). The consistency of the antibody-positivity rate differed significantly between the 2017/2018 and 2019/2020 seasons (*p* = 0.002), but no significant difference was noted compared with that in the 2020/2021 season (*p* = 0.25) (Table 5). The GMT was the highest (44.3) in the 2019/2020 season (Table 6).

## 4. Discussion

The HAI titer of the serum is related to antigenically similar viruses, and the back-boost response is considered to decrease depending on the antigenic distance [15]. In addition, if the difference in the HAI titer is below a fold dilution, the influenza virus is considered to be antigenically similar [16].

For influenza A (H1N1), the antibody titers did not differ between the 2017/2018 and 2019/2020 seasons; the antibody titers in the 2020/2021 and 2021/2022 seasons differed from those in the 2017/2018 and 2019/2020 seasons. There was a large antigenic shift in the strain in the 2020/2021 season and, thereafter, this affected the antibody titer. Furthermore, according to the surveys from the 2017/2018 to 2021/2022 seasons conducted by the National Epidemiological Surveillance of Vaccine-Preventable Diseases (NESVPD) [10,11,12,13], the antibody-positivity rate was high in those aged 5–24 years, but the overall positivity rate decreased with progression of the epidemic season, which is consistent with the results of the present study. In addition, no difference was observed in the positive/negative assessment using the McNemar test. This may have been due to the low positivity rate of the samples. Therefore, it is preferable to evaluate the consistency of the antigenicity of influenza A (H1N1) subtypes in an age group with a high antibody-positivity rate.

For influenza A (H3N2), the antibody titers against the vaccine strain differed with the season. Additionally, the antibody-positivity rate was high in both the 2017/2018 and 2020/2021 seasons, but the consistency of antibody positivity between seasons was low. This suggests that there were wide differences in antigenicity among all the strains of influenza H3N2 evaluated. According to NESVPD [10,11,12,13], the antibody-positivity rate was high in individuals aged 13–40 years. The antibody-positivity rate was lower in all age groups in the 2020/2021 season than in the 2017/2018 season. The NESVPD survey results are consistent with our study results. For influenza A (H3N2), antigenic variation is common if the virus is cultured using eggs [17]. This may also contribute to the differences in antigenicity.

For the influenza B/Victoria lineage, despite changes in the vaccine strain since the 2017/2018 season, no difference was observed in the antibody titer or consistency between the 2017/2018 and 2020/2021 seasons, suggesting small differences in antigenicity. According to NESVPD [10,11,12,13], the positivity rate was also high in the 2019/2020 season. In the 2020/2021 and 2021/2022 seasons, the antibody-positivity rate was high in the 40–59-year age group compared with that in the other age groups.

For the influenza B/Yamagata lineage, the same vaccine strain has been used since the 2017/2018 season. Therefore, we did not compare the strains according to season in this study. According to NESVPD [10,11,12,13], the antibody prevalence exceeded 70% in those aged 25–34 years in the 2021/2022 season.

In the United States, 5–15% of the population generally develops influenza in an influenza epidemic [8]. In Japan, before the COVID-19 pandemic, the estimated number of cases was approximately 7.3 million in the 2018/2019 season and approximately 12 million, which is approximately 10% of the population, in the 2017/2018 season [4,5]. There was a marked reduction in the incidence of influenza in Japan in the 2020/2021 and 2021/2022 seasons, owing to the effect of the COVID-19 pandemic [2]. If there is no exposure to seasonal infections, including influenza, immunity may be reduced and susceptibility to infection may be enhanced [18]. There is, thus, the potential for a large influenza epidemic. The back-boost response to infection is similar to that of vaccination [16]. For this reason, if the strain is similar to that in the previous season, an increase in the number of susceptible individuals may be managed by influenza vaccination.

The incidence rates of many infectious diseases have been reduced by preventive measures practiced during the COVID-19 pandemic [18]. However, a decrease in the infection rate leads to an increase in the number of susceptible individuals, and an abnormal increase in the incidence of respiratory syncytial virus infection has been reported [19]. If infection prevention measures are relaxed, the incidence of infectious diseases that are currently suppressed may increase.

As a seropositive titer, the HAI titer is used as the gold standard [8]. It is also used in the EMA Guidance [9]. In reality, however, an HAI titer of 1:40 represents a level at which 50% of people are protected, and it does not ensure protection from infection [8]. Therefore, it is necessary to simultaneously examine not only the HAI titer (humoral immunity) but also cellular immunity to evaluate immunity against influenza. Inactivated vaccines do not induce cellular immunity [20]. However, we have developed a simple method to evaluate cellular immunity based on the measurement of the interferon-γ level and have shown that inactivated vaccines induce cellular immunity [21]. Furthermore, we showed that there are differences in the estimated timing of the development of cellular immunity, depending on the method of measurement used [22].

The effectiveness of seasonal influenza vaccines may be affected by mismatch with the circulating influenza virus [20]. The vaccine is highly effective when it is similar to the circulating virus [23]. The vaccine may also be effective, to a limited extent, even if its matching is insufficient [24]. In the 2017/2018 season, the majority of the influenza A(H1N1) isolates tested were antigenically similar to the vaccine strain used in Japan and to the WHO-recommended vaccine strain. The antigenic analysis indicated that 50–60% of the influenza A(H3N2) isolates tested were antigenically similar to the cell-grown reference virus influenza A(H3N2) (the vaccine strain for the 2017/2018 season). Among the influenza B/Victoria isolates analyzed, although 90% were antigenically similar to influenza B/Texas/2/2013 (the vaccine strain for the 2017/2018 season) until January 2018, the antigenic similarity dropped to 60% from February 2018 [3]. The antigenic analysis also revealed that almost all the influenza A(H3N2) strains tested were antigenically different from the vaccine strain for the 2019/2020 season. The B/Yamagata lineage was analyzed, and its strains were found to be antigenically similar to the vaccine strain for the 2019/2020 season [5].

A limitation of this study is that the serum collected at one point in the 2017/2018 season was used; thus, the effect of vaccination could not be evaluated. For the future, the evaluation of whether changes in vaccine strains affect cross-immunity will become possible by conducting analyses before and after vaccination, as in this study. As the age of participants in this study varied widely from 29 to 63 years, it is important to conduct an evaluation for narrower age groups. However, adjustment for age was not performed in this study because of the small sample size. We plan to study the effects of age on influenza antibody titers in future studies. Furthermore, as there were seasons with low positivity rates, it is preferable to study age groups with a high antibody-positivity rate. Positivity rates and GMTs were increased for influenza B/Victoria (2019/2020 season) and influenza A (H3N2) (2020/2021 season) compared with those for the corresponding influenza strains in the 2017/2018 season vaccine. This may be due to antigenic closeness to previous epidemic strains. To evaluate this, previous epidemic strains must be examined.

## 5. Conclusions

Because of the preventive measures against COVID-19, the incidence of influenza has remained low since the 2020/2021 season. The results of the present study suggest the potential for marked changes in the antigenicity of seasonal influenza. Additionally, a decreased incidence of influenza leads to an increase in the number of susceptible individuals; therefore, relaxation of preventive measures against COVID-19 may trigger a large influenza epidemic. Evaluation of the antibody titers and their consistency in each season using the same serum enables the evaluation of cross-immunity to different influenza strains.

## Figures and Tables

**Table 1:** Strains used for influenza vaccines in Japan according to season.

Influenza Type	2017/2018 Season	2018/2019 Season	2019/2020 Season	2020/2021 Season	2021/2022 Season
Influenza A (H1N1)	A/Singapore/ GP1908/ 2015(IVR-180)	A/Singapore/ GP1908/ 2015(IVR-180)	A/Brisbane/02/ 2018(IVR-190)	A/Guangdong-Maonan/SWL1536/ 2019(CNIC-1909)	A/Victoria/1/ 2020(IVR-217)
Influenza A (H3N2)	A/Hong Kong/4801/ 2014(X-263)	A/Singapore/ INFMH-16-0019/ 2016(IVR-186)	A/Kansas/14/ 2017(X327)	A/Hong Kong/2671/ 2019(NIB-121)	A/Tasmania/503/ 2020(IVR-221)
Influenza B/Yamagata	Phuket/3073/2013	Phuket/3073/2013	Phuket/3073/2013	Phuket/3073/2013	Phuket/3073/2013
Influenza B/Victoria	Texas/2/2103	B/Maryland/15/2016 (NYMC BX-69A)	B/Maryland/15/2016 (NYMC BX-69A)	B/Victoria/705/ 2018(BVR-11)	B/Victoria/705/ 2018(BVR-11)

**Table 2:** Spearman’s correlation coefficients comparing the antibody titers in other seasons with those in the 2017/2018 season.

Influenza Type	2019/2020	2020/2021	2021/2022
Influenza A (H1N1)	0.90	0.50	0.38
Influenza A (H3N2)	0.59	0.71	0.70
Influenza B/Victoria	0.91	0.83	… ^†^

^†^ Same as in the previous season.

**Table 3:** Wilcoxon signed-rank test results assessing the statistical significance of differences in antibody titers compared with those in the 2017/2018 season.

Influenza Type	2019/2020	2020/2021	2021/2022
Influenza A (H1N1)	*p* > 0.99	*p* = 0.002 *	*p* = 0.014 *
Influenza A (H3N2)	*p* < 0.001 *	*p* < 0.001 *	*p* < 0.001 *
Influenza B/Victoria	*p* < 0.001 *	*p* = 0.17	… ^†^

^†^ Same as the previous season. * *p* < 0.05.

**Table 4:** Percentage of participants with hemagglutination inhibition titers ≥1:40.

Influenza Type	2017/2018	2019/2020	2020/2021	2021/2022
Influenza A (H1N1)	5/34 (15%)	5/34 (15%)	1/34 (3%)	1/34 (3%)
Influenza A (H3N2)	20/34 (59%)	3/34 (9%)	29/34 (85%)	2/34 (6%)
Influenza B/Victoria	12/34 (35%)	22/34 (65%)	9/34 (26%)	… ^†^

^†^ Same as in the previous season.

**Table 5:** Results of McNemar test assessing the consistency of changes in antibody positivity compared with those in the 2017/2018 season.

Influenza Type	2019/2020	2020/2021	2021/2022
Influenza A (H1N1)	*p* > 0.99	*p* = 0.13	*p* = 0.22
Influenza A (H3N2)	*p* < 0.001 *	*p* = 0.003 *	*p* < 0.001 *
Influenza B/Victoria	*p* = 0.002 *	*p* = 0.25	… ^†^

^†^ Same as in the previous season. * *p* < 0.05.

**Table 6:** Geometric mean titers of antibodies to vaccine strains in different seasons.

Influenza Type	2017/2018	2019/2020	2020/2021	2021/2022
Influenza A (H1N1)	10.4	11.5	6.0	5.9
Influenza A (H3N2)	30.7	12.0	58.9	9.0
Influenza B/Yamagata	25.0	… ^†^	… ^†^	… ^†^
Influenza B/Victoria	17.7	44.3	15.0	… ^†^

^†^ Same as in the previous season.

## Data Availability

The data presented in this study are available on reasonable request from the corresponding author.
